# Supplementary material for: Prevalence and Predictors of Food Insecurity among Students of a Spanish University during the COVID-19 Pandemic: FINESCOP Project at the UPV/EHU
Source: Nutrients. 2023 Apr 11;15(8):1836. doi: 10.3390/nu15081836 (PMC10142759; doi:10.3390/nu15081836)
Supplement: Supplementary file 1 [file nutrients-15-01836-s001.zip › Supplementary Table S1_Nutrients_.pdf]

Supplementary Table S1. Findings of other studies on the prevalence of food insecurity among students of higher education, before and during the COVID-19 pandemic.

Author (ref.)	Study design	Date of data collection	Country	Sample size	Assessment	FI rate	Pre/during the COVID-19 pandemic
Patton-López et al. [12]	Cross-sectional	May 2011	USA	354	Six-item USDA-FSSM	59%	Pre
Raskind et al. [38]	Longitudinal cohort	October/November 2014, February/March 2015, June/ July 2015, and October/November 2015	USA	2377	Six-item USDA-FSSM	29%	Pre
El Zein et al. [11]	Cross-sectional	Fall 2015, and Spring 2016 academic semesters	USA	855	Ten-item USDA-FSSM	19%	Pre
Theodoritis et al. [20]	Cross-sectional	2016	Greece	236	HFIAS	82.1%	Pre
Adamovic et al. [13]	Cross-sectional	December 2016, and February 2017	USA	339	Six-item USDA-FSSM	54%	Pre
Sackey et al. [39]	Cross-sectional	2019	USA	302	Six-item USDA-FSSM	28.2%	Pre
Ahmad et al. [40]	Cross-sectional	May 2019 to January 2020	Malaysia	663	Six-item USDA-FSSM	62.8%	Pre
Moore et al. [10]; Owens et al. [14]	Cross-sectional	May 2020	USA	651	Two-item Food Sufficiency Screener, and six-item USDA-FSSM	34.5%	During
Mihrshahi et al. [23]	Cross-sectional	July and September 2020	Australia	105	Six-item USDA-FSSM	41.9%	During
Marques et al. [22]	Cross-sectional	December 2021 to February 2022	Portugal	284	Brazilian Food Insecurity Scale (an adaption of the USDA-FSSM)	17.3%	During

Abbreviations: FI, food insecurity; HFIAS, Household Food Insecurity Access Scale; USDA-FSSM, United States Department of Agriculture Food Security Survey Module.
