# Supplementary material for: Prevalence and Predictors of Food Insecurity among Students of a Spanish University during the COVID-19 Pandemic: FINESCOP Project at the UPV/EHU
Source: Nutrients. 2023 Apr 11;15(8):1836. doi: 10.3390/nu15081836 (PMC10142759; doi:10.3390/nu15081836)
Supplement: Supplementary file 1 [file nutrients-15-01836-s001.zip › Supplementary Table S2_Nutrients_.pdf]

Supplementary Table S2. Population and sample of the University of the Basque Country UPV/EHU by field of education and age.

Field of education	Age	UPV/EHU's population [1]		Real sample		Weighting coefficient	Weighted sample,
		n	%	*n*	%		*n*
Education	18	573	12.7	15	15.5	38.2	573
	19	667	14.8	12	12.4	55.6	667
	20	771	17.1	13	13.4	59.3	771
	≥21	2506	55.5	57	58.8	44.0	2506
	Total	4517		97 [2]			4517
Arts and Humanities	18	627	12.3	2	4.4	313.5	627
	19	716	14.1	2	4.4	358	716
	20	744	14.6	12	26.7	62	744
	≥21	2994	58.9	29	64.4	103.2	2994
	Total	5081		45 [2]			5081
Social Sciences, Journalism, and Information	18	645	11.4	20	19.0	32.3	645
	19	770	13.6	28	26.7	27.5	770
	20	795	14.1	13	12.4	61.2	795
	≥21	3438	60.9	44	41.9	78.1	3438
	Total	5648		105 [2]			5648
Business, Administration, and Law	18	1114	14.6	1	3.6	1114	1114
	19	1076	14.1	4	14.3	269	1076
	20	1043	13.7	4	14.3	260.8	1043
	≥21	4385	57.6	19	67.9	230.8	4385
	Total	7618		28 [2]			7618
Natural Sciences, Mathematics, and Statistics	18	543	14.7	10	16.7	54.3	543
	19	513	13.9	4	6.7	128.3	513
	20	509	13.8	8	13.3	63.6	509
	≥21	2135	57.7	38	63.3	56.2	2135
	Total	3700		60 [2]			3700
Information, and Communication	18	142	20.7	4	26.7	35.5	142
	19	143	20.8	3	20.0	47.7	143
	20	80	11.7	3	20.0	26.7	80
	≥21	321	46.8	5	33.3	64.2	321
	Total	686		15 [2]			686
Engineering, Manufacturing, and Construction	18	1415	14.0	5	13.5	283	1415
	19	1141	11.3	2	5.4	570.5	1141
	20	1174	11.6	5	13.5	234.8	1174
	≥21	6348	63.0	25	67.6	253.9	6348
	Total	10,078		37 [2]			10078
Health and Welfare	18	784	13.2	3	8.6	261.3	784
	19	819	13.8	3	8.6	273	819
	20	841	14.2	3	8.6	280.3	841
	≥21	3477	58.7	26	74.3	133.7	3477
	Total	5921		35 [2]			5921
Services	18	88	11.4	0	0	-	0
	19	91	11.8	0	0	-	0
	20	103	13.4	0	0	-	0
	≥21	489	63.4	0	0	-	0

	Total	771	0 [2]	0
Total		44,020	422 [3]	43,249

Note: [1] The sample was drawn from the matriculation list of the UPV/EHU during 2021/22 (data provided by the Vice-rectorate of Digital Transformation and Communication of the UPV/EHU); [2] Participation rate by each field of education; [3] Participation rate in the total sample.
